# Mild Treatment of Scarlatina

**Published:** 1854-03

**Authors:** Joseph Miller

**Affiliations:** Missouri


### RECORD OF MEDICAL SCIENCE.

*Mild Treatment of Scarlatina.* By JOSEPH MILLER, M. D., *of Missouri.* —An epidemic of Scarlatina variable in intensity has prevailed during the latter part of autumn and beginning of winter, throughout the counties bordering on the Missouri River, from St. Louis to Council Bluffs It has presented in the same localities, the simple, anginose and malignant varieties, but with less tendency to recession of the eruption and local complications than usually attends the disease. It is not with the intention of presenting anything new upon this interesting subject, that I send you this very brief communication, but merely to add my testimony and experience to those former observations which encourage a mild and simple course of medication in the disease. It is very certain, that fewer fatal cases have fallen under my notice during the past year, than for twenty-five years previous, and yet the disease has exhibited, in a majority of cases its usual intensity and persistence. I attribute this, not to anything in its nature or pathology, from what is usually observed, but to the fact, that a milder treatment has been gaining favor with the profession in my range of observation. I do not wish to find fault, or avow any peculiar skill in the treatment of disease, but I believe half the mortality of scarlet fever as reported from some localities, has been owing to the severe antiphlogistic treatment pursued. Scarlatina is a specific disease, and as such, besides other peculiarities, has a definite course to run. The rise, progress and decline are strictly defined by certain conditions of the system. It is not anything peculiar to this disease that excites the tendency to complications and sequela, which prove so obstinate and dangerous. All diseases which deprave the blood manifest the same tendency. It is when the culmination and decline of the disease is interrupted and turned from the ordinary course, that these frightful accidents supervene. There is a harmony in the various operations by which morbid impressions are opposed and finally extruded from the economy, as well as in the physiological combinations; and it is reasonable to suppose that this harmony adapts itself to the various ends to be accomplished. For example in many diseases (Scarlatina for one) the great burden of elimination is thrown upon the kidneys. The act of depuration in this case is a healthy one and calculated to restore health to the system; interrupt it by a strong impression of medicine on distant but correlative organs, or depress the entire organism by undue depletion, and you at once close the principal exit of these diseases from the blood, and retained in the system, it first manifests its evil effects upon the organs where a plus of vitality was instituted for good purposes, and then spreads to other parts.

There are other explanations, but it is well in such investigations to separate true pathology and facts, from hypothesis and probabilities, and to this we contribute by associating, as far as possible, pathological conditions with the known effects of therapeutic agents and our practical observation.

Entertaining these views, I have been led to reject those remedies in scarlet fever, which by depressing the energies of the frame, or in causing the depravation of the blood, or setting up a revulsion, or counter-irritant action on different parts, arrest or divert the efforts at elimination. When disease invades the frame, there are but two ways to get rid of it: 1st, to neutralize it in the body; 2d, to drive it out through the various emunctories. The first cannot be done in scarlet fever. It therefore becomes necessary to aid the process of elimination by such gentle means as will not excite in the secernent organs engaged, too much local hyperemia. For this purpose, I employ, in every variety of scarlatina, simple, anginose or malignant, the mildest diuretics and diaphoretics; such as Cream of Tartar, Spirits of Nitre, Chlorate of Potash, &c. It is no contradiction to these views to employ tonics or stimulants in the severe examples of the malignant disease, for we thereby inspire a necessary energy in the depurative organs, which other means, unaided, would fail to excite.

This course, with such attention to the skin, temperature, and diet, as every physician's experience will suggest, constitutes our treatment of scarlet fever, and our observation enables us to recommend it to the profession.—*West. Medico-Chirurgical Journal.*

